# How to Remove Excess of Varnish from Cavities

**Published:** 1900-02-15

**Authors:** 


### HOW TO REMOVE EXCESS OF VARNISH FROM CAVITIES.

After varnishing a cavity, and before introducing the filling, remove excess of varnish by using small pieces of rubber-dam. It takes up the varnish and leaves no lint behind.—*Australian Journal of Dentistry.*

